# Top cited articles in Oral Radiology: A bibliometric network analysis

**DOI:** 10.4317/jced.61730

**Published:** 2024-07-01

**Authors:** Anastasia Fardi, Konstantinos Kodonas, Theodoros Lillis, Antigoni Delantoni, Nikolaos Dabarakis

**Affiliations:** 1 Assistant Professor, Department of Dentoalveolar Surgery, Surgical Implantology & Radiology, School of Dentistry, Aristotle University of Thessaloniki, Greece; 2 Assistant Professor, Department of Endodontology, School of Dentistry, Aristotle University of Thessaloniki, Greece; 3 DDS, Associate Professor, Department of Dentoalveolar Surgery, Surgical Implantology & Radiology, School of Dentistry, Aristotle University of Thessaloniki, Greece

## Abstract

**Background:**

The present study aimed to identify and analyze the 100 top-cited articles published in oral radiology.

**Material and Methods:**

Web of Science was used to conduct a comprehensive search from inception until 22 November 2023 in dental radiology. Basic information of the 100 top-cited articles was recorded. Biblioshiny and VOSviewer tools were employed for conducting thematic map and author keyword, title, and abstract terms analysis to elucidate the research trends and hotspots. Elsevier Scopus database was also used for citation comparisons.

**Results:**

The citation count for the 101 most-cited articles ranged from 105-587. Most of them were original research studies with observational design conducted in diagnosis, dose, geometric measurements, and image analysis topics. Cone beam computed tomography was the most studied radiologic technique as author keyword co-occurrence analysis revealed and appeared as a basic theme for the transdisciplinary research field’s development. While making infant steps, artificial intelligence was adequately represented in top cited list, as it received increasing citation numbers in very few years, concentrating the highest citation densities.

**Conclusions:**

Bibliometric analysis of the most affecting publications in oral radiology depicts the science’s evolution and enhances the understanding of scientific research progress.

** Key words:**Bibliometrics, citation analysis, oral radiology, top-cited.

## Introduction

Bibliometric analysis combines science with statistical mathematics in order to describe trends of knowledge within a research field and provide an in depth perceptive of scientific literature evolution (1). As a statistical tool, it facilitates the understanding and evaluation of the scientific performance and impact of a paper, as well as the recognition of prominent authors, countries and institutions within a specific scientific community (1,2).

Many general or specialized fields of dentistry have been analysed through bibliometric tools and citation analysis software (3-7). Besides the scarcity of such an analysis in oral radiology, some available data have been published within the field of diagnostic radiology (8-11). Two citation analyses had been conducted in 2018 investigating mainly the applications of oral and maxillofacial cone beam computed tomography (CBCT) (12,13).

To the best of our knowledge, citation analysis has not been performed within oral radiology field, so far. The aim of the present study was to to identify the characteristics of the top-cited articles published in oral radiology to explore the dynamic research trends, provide insights into discipline’s evolution and define influential papers, prolific researchers, organizations and countries.

## Material and Methods

Journal Citation Reports 2023 (JCR; Thomson Reuters, New York, NY) was utilized to identify dental radiology journals (14). In the Web of Science (WoS) database 4 journals were considered as the most eligible specifically pertaining to oral radiology: Dentomaxillofacial Radiology (IF=3.3), Oral Surgery Oral Medicine Oral Pathology Oral Radiology (IF= 2.9), Imaging Science in Dentistry (IF=1.8) and Oral Radiology (IF=2.2). Using the above mentioned publication titles; an electronic search was conducted on 22 November 2023. No restriction was applied regarding publication year or study design. The search provided a list of 101 publications ranked by times citation. Elsevier’s Scopus was also used to crossmatch the citation counts of the top-cited papers.

Two study investigators reviewed independently and assessed the articles for appropriateness for inclusion. In cases of disagreement, a third reviewer resolved any difference of opinion by consensus review. As long as the “Oral surgery Oral Medicine Oral Pathology Oral Radiology” is not fully dedicated to radiology, only relevant articles were considered in citation analysis. The search provided a list of 100 publications appearing in the radiology journals ranked by citation count. Elsevier’s Scopus was also used to crossmatch the citation counts of the top cited papers. Basic information of the top-cited articles was recorded including number of citations, publication year, publishing journal, authorship, institution and country of origin, type of article, radiologic technique, study design and field of study. The type of article was classified as original, review, position paper, editorial, systematic review, and meta-analysis. According to study design, original scientific papers were further categorized into basic science and prospective or retrospective observational studies. Articles were also categorized according to the radiologic technique and topic. Topic and research trends analysis was based on the high-frequency keywords, abstract and title terms of each included paper. Software VOSviewer (version 1.6.11) and R package “Bibliometrix” was used for co-occurrence network (15,16) and science mapping analysis (17).

## Results

-Citations, publication sources and years

 Table 1 summarizes the 100 most cited articles published in dental radiology journals, by order of decreasing number of citations. The most cited article received 587 (WoS) and 648 (Scopus) citations, and the least-cited paper counted 105 and 86 citations (WoS and Scopus), respectively. There were 21 articles in WoS and 24 articles in Scopus that received more than 200 citations. The most represented journal was “Oral surgery Oral Medicine Oral Pathology Oral Radiology”, containing 52 highly cited articles, followed nearly by “Dentomaxillofacial Radiology” (48 articles).

**Figure 1:**
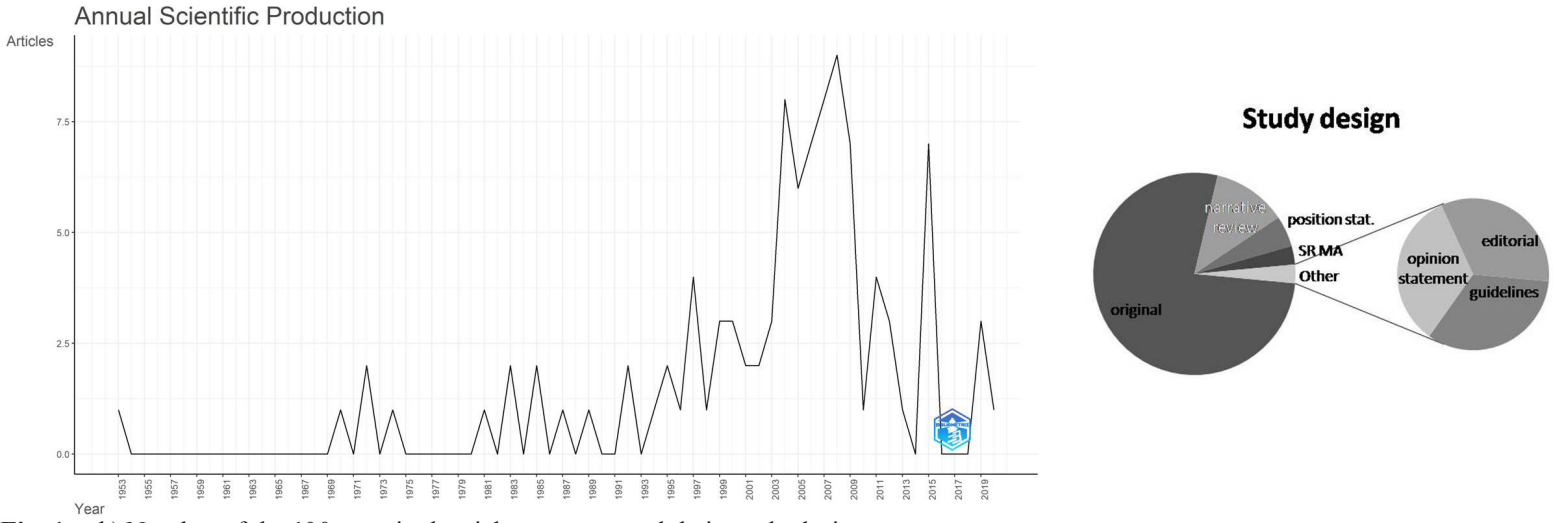
a,b) Number of the 100 top-cited articles per year and their study design.

Figure 1a shows the distribution of the 100 articles by year of publication. All articles were published from 1953 until 2020, with a publication peak between 2004 and 2009. 2008 and 2007 were the most productive years publishing 9 and 8 most cited papers, respectively. Through the previous decades, the publication race was stable.

-Country of origin, institution, and authorship pattern 

According to the institutional address of the corresponding author, researchers from 21 countries contributed the 101 top-cited papers, with USA publishing the largest number of them (n=40). The University of North Carolina in USA was also the most productive institution. A total of 367 different researchers authored highly cited papers, with 17 publishing at least 3 articles. Six “frequent authors” held authorship for more than 4 papers. Relative data regarding countries, institutions and authors with the highest number of manuscripts are presented in  Table 2.

-Study design, radiologic technique.

Most of the most-cited papers considered original research (n=78), followed distally by narrative reviews (n=12), position statements(n=5), systematic reviews and meta-analyses(n=3) and others (n=3) (Fig. 1b). Original research papers were further classified as having observational study design (n=44) with retrospective (n=21), prospective (n=13) approach, case reports (n=6), cross-sectional (n=5) and basic science content (n=33). Major topics of interest covered in the top-cited list are presented in  Table 3. Most top-cited papers investigated several aspects of CBCT.

-Field of study, author keyword, title and abstract term analysis 

**Figure 2:**
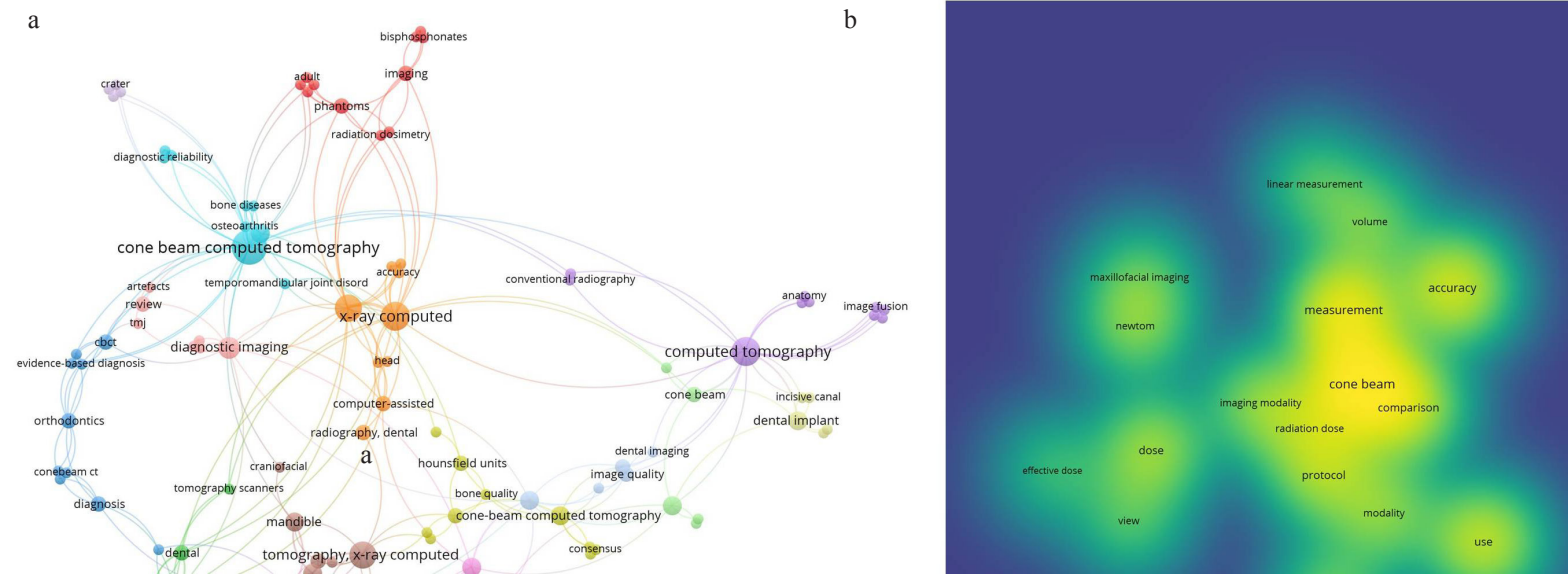
a. Co-occurrence network map of author keywords. Each node represents a keyword in the network and its size is directly proportional to its number of occurrences in the including papers. Colors indicate clusters to which keywords are assigned together. b. Term network map extracted from the title and abstract fields of the top-cited publications.

**Figure 3:**
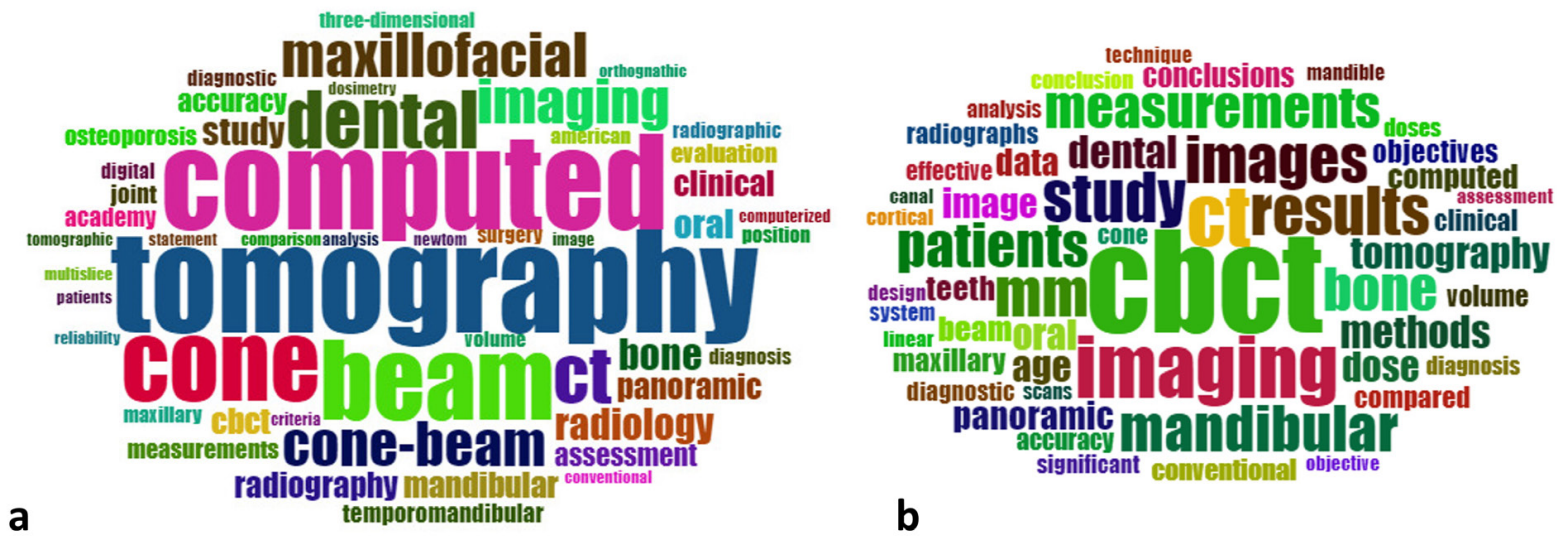
WordCloud analysis of top-cited publications’ title (a) and abstract (b) terms.

Author keyword co-occurrence analysis indicated cone beam computed tomography (n=10) as the most prevalent term, followed by x-ray computed (n=7) (Fig. 2a). Combining title and abstract fields in VOSviewer, cone beam (n=27), measurement (n=22), accuracy (n=19) and diagnosis (n=19) were commonly used as depicted in Fig. 2b. According to WordCloud analysis, tomography and computed were commonly used as title terms (Figure 3a), while CBCT was frequently recorded in abstract terms (n=169), followed by CT (n=102) and imaging (n=101) (Fig. 3b).

**Figure 4:**
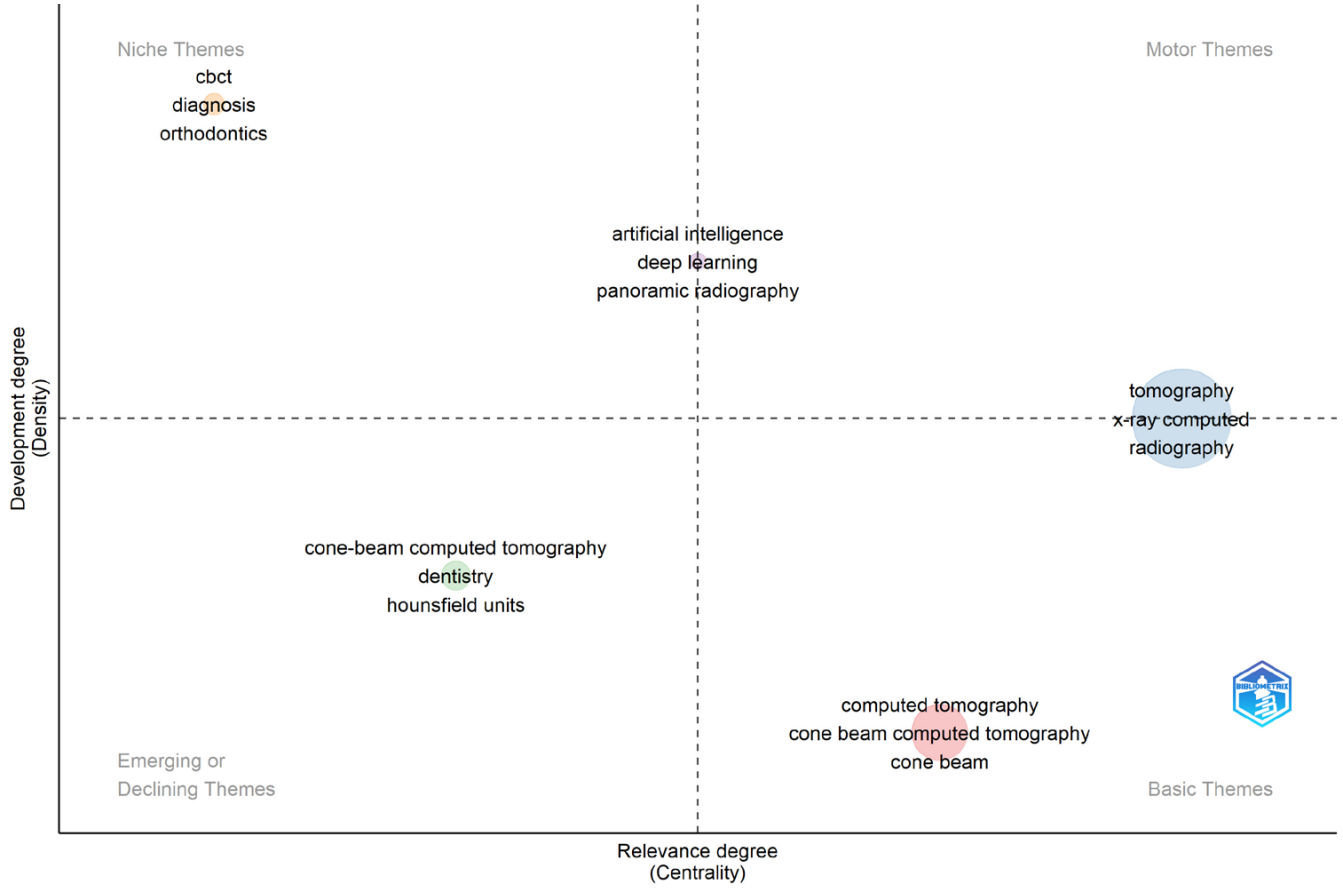
Thematic map analysis of top cited author keywords.

Figure 4 displays the results of the thematic map analysis of author keywords. “CBCT”, “diagnosis”, and “orthodontics” were identified as niche themes in the top left quadrant (Q2). “Artificial intelligence”, “deep learning” and “panoramic radiography” centrally located with high centrality and medium density were sandwiched as niche (Q2) and motor themes (Q1), simultaneously. In the same way, “tomography”, “x-ray computed” and “radiography” was found to be motor (Q1) and basic theme (Q4), concurrently. The lower right quadrant (Q4) included basic central themes with clusters of “cone beam”, “computed tomography” and “cone beam computed tomography”. Cluster of “Housefield units” in “dentistry” was identified as a declining theme in the lower left quadrant (Q3).

## Discussion

Oral and maxillofacial radiology has been evolved into an innovative and rapidly expanding field of imaging concentrating several important achievements, following technological advancements. As far as the authors are aware, this is the first citation analysis in oral and maxillofacial radiology. The number of citations for the 101 top-cited articles presented herein ranged from 105 to 587 (WoS) or 86 to 648 (Scopus) during the period 1953-2020.

According to the results of the present report, top 4 contributing countries included USA, Japan, Germany, and Belgium. It is notable here that the first two countries, contributed 40 and 13 manuscripts, respectively, consisting 53% of the sample. As evidenced in the present citation analysis and in accordance with previous investigations, USA led the top-cited list (5-7,9). This could be attributed to the large community of active researchers and sufficient finances for conducting health science research. In addition, a tendency of U.S. authors to cite local papers along with the recorded preference of U.S. reviewers for U.S. manuscripts has been documented and could explain USA dominance (18,19). Consistently, top institution also originated from USA, producing 11 top-cited articles during the period 1997–2015.

As it was expected, 78 highly cited papers included original research, with 45 of them having observational study design and 33 being published within the basic science field. The dominance of original research in oral radiology results from the specialty’s nature and indicates the inclination of many researchers to integrate the existing information and knowledge of basic studies into clinical practise. No randomized clinical trial (RCT) was included in the top-cited list. Considering the time depended requirements in meeting the demands of highly evidenced RCTs along with concerns prohibiting comparison of radiographic modalities in humans, documentation of clinical approaches to oral radiology by observational studies offers treatment modalities directly in less time.

This study demonstrated a slight preeminence of clinical rather than basic science articles, a trend that has already been evidenced in various disciplines of clinical dentistry (6,7). Contradictory results had been recorded in medical imaging literature, with preclinical studies being highly represented in the top-cited list (8). The previous citation analysis in CBCT exhibited the predominance of basic-technical studies, which was attributed to the fact that imaging research is highly dependent on advances in other fields such as computer science and physics (13).

Indeed, basic preclinical radiologic research represents an important chapter in radiology for conducting dosimetry studies and evaluating the effectiveness of new modified techniques and modalities. Similar to our study, Brinjikji and colleagues demonstrated that, six of the top-cited articles presented major technical advancements by introducing new techniques or technologies (8). Interesting observations arisen from the inclusion of 5 position papers in the top-cited list, 1 editorial paper and 1 opinion statement. This reveals the urgent need of establishing clinical therapeutic protocols on dental radiology by utilizing knowledge from new techniques presented worldwide and reflects a citation analysis characteristic, named as “snowball effect”, where researchers tended to cite previous highly cited articles (20). Moreover, the inclusion of 3 systematic reviews and one meta-analysis among the top-cited publications designated research allocation towards evidence based dentistry.

CBCT was the most prevalent radiologic technique among the top-cited list, a fact that was also evidenced in the abstract term co-occurrence analysis and indicative of this innovative radiographic modality’s constant research evolution for oral and maxillofacial imaging tasks. It should be noted here that the majority of studies dealing with CBCT were published from 1999 until 2015, which is consistent with the emergence of the CBCT use in oral and maxillofacial region in the beginning of the 21st century.

Taken all the research topics together, linear and dose measurements, osteoporosis, presentations of new techniques and modalities as well as TMJ and impacted teeth imaging occupied the leading positions in the top-cited list, a fact indicative of the importance of the above topics in dental community. Artificial intelligence, an emerging applied computer software science was an adequately represented research field in the top-cited papers concentrating the highest citation density values, despite its recent development, which points out the critical driving power and prevailing capabilities of AI in data analysis. In spite of its infant steps, AI has emerged in dentistry and has heralded an era of disruptive technology with the potential to play assistive and supplementary role to medical community (21).

Regarding the thematic map analysis using author keywords, “CBCT” uses in “orthodontic” “diagnosis” were represented as niche themes with high density and low centrality, signifying that they are nonetheless very specialized of rapid development topics. Notably from the Figure, themes such as tomography, x-ray computed and radiography sandwiched between motor and basic themes are well developed and capable of structuring the research field. The left bottom quadrant contains “cone beam computed tomography”, “Housefield units” in “dentistry” that have been adequately developed and yet have experienced a downward trend, as indicated by low centrality and density “AI, deep learning and panoramic radiography” appear to transverse from niche to motor themes and tend to become more central essential for the understanding but still of marginal contribution to the development of oral radiology field. Finally, themes such as “cone beam computed tomography” seen as basics are very important for transdisciplinary research field’s development.

Regarding the use of specific databases, an appropriate source should be selected to have accurate and reliable data analysis. Citation indexes like WoS databases’, mainly focus on journals and less on other means of scientific knowledge diffusion (books and proceedings). As articles published in non WoS-cited journals are excluded, it is still unclear whether WoS represents the best available tool that could be used for research evaluation. Both databases WoS and Elsevier’s Scopus were included for the citation calculations to overcome methodological limitations, as citation counts have been shown to differ significantly between these two databases.

This study identified and analyzed the top-cited articles in dental radiology journals to describe the research trends and progress of this rapidly evolving field of dentistry. Identification of top cited list may be of substantial interest to dental clinicians providing an auxiliary guide for educational and training purposes. Within its limited scope, the results of the present citation analysis provide a historical perspective on evolution of oral radiology discipline and highlight the most impactful authors, institutions, and countries. Original studies with observational study design conducted in diagnosis were the mainstay of the top-cited papers in oral and maxillofacial radiology. The United States ranked first with the highest number of top-cited publications. Understanding the characteristics of highly cited articles in imaging literature of dental radiology journals emphasizes important advancements achieved in this field.

## Figures and Tables

**Table 1:** The 101 top cited articles in Oral radiology.

Rank	Article	WoS*	Scopus*
1	Schulze R, Heil U, Gross D, Bruellmann D, Dranischnikow E, Schwanecke U, et al. Artefacts in CBCT: a review Dentomaxillofac Radiol 2011;40:265-73.	587 (48,92)	648 (58,91)
2	Ludlow JB, Davies-Ludlow LE, Brooks SL, Howerton WB. Dosimetry of 3 CBCT devices for oral and maxillofacial radiology: CB Mercuray, NewTom 3G and i-CAT Dentomaxillofac Radiol 2006;35:219-26.	532 (31,29)	605 (35,59)
3	Ludlow JB, Ivanovic M. Comparative dosimetry of dental CBCT devices and 64-slice CT for oral and maxillofacial radiology. Oral Surg Oral Med Oral Pathol Oral Radiol Endod 2008;106:106-14.	512 (34,13)	659 (43,93)
4	Jaffe HL. Giant-cell reparative granuloma, traumatic bone cyst, and fibrous (fibro-osseous) dysplasia of the jawbones. Oral Surg Oral Med Oral Pathol Oral Radiol Endod 1953;6:159-175.	495 (7,07)	496 (7,08)
5	Arai Y, Tammisalo E, Iwai K, Hashimoto K, Shinoda K. Development of a compact computed tomographic apparatus for dental use. Dentomaxillofac Radiol 1999;28:245-8.	455 (18,96)	535 (22,29)
6	Marx RE, Johnson RP. Studies in the radiobiology of osteoradionecrosis and their clinical significance. Oral Surg Oral Med Oral Pathol 1987;64:379-90.	440 (12,22)	506 (14,06)
7	Ahmad M, Hollender L, Anderson Q, Kartha K, Ohrbach R, Truelove EL, et al. Research diagnostic criteria for temporomandibular disorders (RDC/TMD): development of image analysis criteria and examiner reliability for image analysis Oral Surg Oral Med Oral Pathol Oral Radiol Endod 2009;107:844-60.	390 (27,86)	449 (32,07)
8	Pineda F, Kuttler Y. Mesiodistal and buccolingual roentgenographic investigation of 7,275 root canals. Oral Surg Oral Med Oral Pathol 1972;33:101-10.	378 (7,41)	503 (9,86)
9	Lascala CA, Panella J, Marques MM. Analysis of the accuracy of linear measurements obtained by cone beam computed tomography (CBCT-NewTom). Dentomaxillofac Radiol 2004;33:291-4.	349 (18,37)	393 (20,68)
10	Grover PS, Lorton L. The incidence of unerupted permanent teeth and related clinical cases. Oral Surg Oral Med Oral Pathol 1985;59:420-5.	302 (7,95)	335 (8,82)
11	Lofthag-Hansen S, Huumonen S, Gröndahl K, Gröndahl HG. Limited cone-beam CT and intraoral radiography for the diagnosis of periapical pathology. Oral Surg Oral Med Oral Pathol Oral Radiol Endod 2007;103:114-9.	290 (18,13)	350 (21,88)
12	Ludlow JB, Davies-Ludlow LE, Brooks SL. Dosimetry of two extraoral direct digital imaging devices: NewTom cone beam CT and Orthophos Plus DS panoramic unit. Dentomaxillofac Radiol 2003;32:229-34.	284 (14,2)	339 (16,95)
13	Tyndall DA, Price JB, Tetradis S, Ganz SD, Hildebolt C, Scarfe WC. Position statement of the American Academy of Oral and Maxillofacial Radiology on selection criteria for the use of radiology in dental implantology with emphasis on cone beam computed tomography. Oral Surg Oral Med Oral Pathol Oral Radiol 2012;113:817-26.	265 (24,1)	298 (27,1)
14	White SC, Rudolph DJ. Alterations of the trabecular pattern of the jaws in patients with osteoporosis. Oral Surg Oral Med Oral Pathol Oral Radiol Endod 1999;88:628-35.	261 (10,88)	276 (11,5)
15	Ludlow JB, Timothy R, Walker C, Hunter R, Benavides E, Samuelson DB, et al. Effective dose of dental CBCT-a meta analysis of published data and additional data for nine CBCT units. Dentomaxillofac Radiol 2015;44:20140197.	249 (31,13)	291 (36,38)
16	Schulze D, Heiland M, Thurmann H, Adam G. Radiation exposure during midfacial imaging using 4- and 16-slice computed tomography, cone beam computed tomography systems and conventional radiography. Dentomaxillofac Radiol 2004;33:83-6.	244 (12,84)	289 (15,21)
17	Mah P, Reeves TE, McDavid WD. Deriving Hounsfield units using grey levels in cone beam computed tomography. Dentomaxillofac Radiol 2010;39:323-35.	226 (17,38)	257 (19,77)
18	Mah JK, Danforth RA, Bumann A, Hatcher D. Radiation absorbed in maxillofacial imaging with a new dental computed tomography device. Oral Surg Oral Med Oral Pathol Oral Radiol Endod 2003;96:508-13.	226 (11,3)	262 (13,1)
19	Cevidanes LH, Bailey LJ, Tucker GR Jr, Styner MA, Mol A, Phillips CL. Superimposition of 3D cone-beam CT models of orthognathic surgery patients. Dentomaxillofac Radiol 2005;34:369-75.	224 (12,45)	260 (14,45)
20	Evans CA, Scarfe WC, Ahmad M, Cevidanes LHS, Ludlow JB, Palomo JM, et al. Clinical recommendations regarding use of cone beam computed tomography in orthodontics. Position statement by the American Academy of Oral and Maxillofacial Radiology. Oral Surg Oral Med Oral Pathol Oral Radiol 2013;116:238-57.	209 (20,9)	234 (23,4)
21	Pinsky HM, Dyda S, Pinsky RW, Misch KA, Sarment DP. Accuracy of three-dimensional measurements using cone-beam CT. Dentomaxillofac Radiol 2006;35:410-6.	205 (12,06)	236 (13,88)
22	Hashimoto K, Arai Y, Iwai K, Araki M, Kawashima S, Terakado M. A comparison of a new limited cone beam computed tomography machine for dental use with a multidetector row helical CT machine. Oral Surg Oral Med Oral Pathol Oral Radiol Endod 2003;95:371-7.	195 (9,75)	228 (11,4)
23	Special Committee to Revise the Joint AAE/AAOMR Position Statement on use of CBCT in Endodontics. AAE and AAOMR Joint Position Statement: Use of Cone Beam Computed Tomography in Endodontics 2015 Update. Oral Surg Oral Med Oral Pathol Oral Radiol 2015;120:508-12.	194 (24,25)	246 (30,75)
24	Pauwels R, Jacobs R, Singer SR, Mupparapu M. CBCT-based bone quality assessment: are Hounsfield units applicable? Dentomaxillofac Radiol 2015;44:20140238.	193 (24,13)	247 (30,88)
25	Johansson B, Grepe A, Wannfors K, Hirsch JM. A clinical study of changes in the volume of bone grafts in the atrophic maxilla. Dentomaxillofac Radiol 2001;30:157-61.	192 (8,73)	191 (8,68)
26	Goldman M, Pearson AH, Darzenta N. Endodontic success-who's reading the radiograph? Oral Surg Oral Med Oral Pathol 1972;33:432-7.	191 (3,75)	235 (4,61)
27	Suomalainen A, Kiljunen T, Käser Y, Peltola J, Kortesniemi M. Dosimetry and image quality of four dental cone beam computed tomography scanners compared with multislice computed tomography scanners. Dentomaxillofac Radiol 2009;38:367-78.	189 (13,5)	212 (15,14)
28	Tsiklakis K, Syriopoulos K, Stamatakis HC. Radiographic examination of the temporomandibular joint using cone beam computed tomography. Dentomaxillofac Radiol 2004;33:196-201.	189 (9,95)	213 (11,21)
29	Ziegler CM, Woertche R, Brief J, Hassfeld S. Clinical indications for digital volume tomography in oral and maxillofacial surgery. Dentomaxillofac Radiol 2002;31:126-30.	189 (9)	218 (10,38)
30	Brooks SL, Brand JW, Gibbs SJ, Hollender L, Lurie AG, Omnell KA, et al. Imaging of the temporomandibular joint: a position paper of the American Academy of Oral and Maxillofacial Radiology. Oral Surg Oral Med Oral Pathol Oral Radiol Endod 1997;83:609-18.	189 (7,27)	223 (8,58)
31	Kapila S, Conley RS, Harrell WE Jr. The current status of cone beam computed tomography imaging in orthodontics. Dentomaxillofac Radiol 2011;40:24-34.	186 (15,75)	216 (18)
32	Tantanapornkul W, Okouchi K, Fujiwara Y, Yamashiro M, Maruoka Y, Ohbayashi N, et al. A comparative study of cone-beam computed tomography and conventional panoramic radiography in assessing the topographic relationship between the mandibular canal and impacted third molars. Oral Surg Oral Med Oral Pathol Oral Radiol Endod 2007;103:253-9.	186 (11,63)	207 (12,94)
33	Pauwels R, Araki K, Siewerdsen JH, Thongvigitmanee SS. Technical aspects of dental CBCT: state of the art. Dentomaxillofac Radiol 2015;44:20140224.	180 (22,5)	251 (31,36)
34	Ludlow JB, Laster WS, See M, Bailey LJ, Hershey HG. Accuracy of measurements of mandibular anatomy in cone beam computed tomography images. Oral Surg Oral Med Oral Pathol Oral Radiol Endod 2007;103:534-42.	180 (11,25)	215 (13,44)
35	Kapila SD, Nervina JM. CBCT in orthodontics: assessment of treatment outcomes and indications for its use. Dentomaxillofac Radiol 2015;44:20140282.	172 (21,5)	215 (26,88)
36	Alexiou K, Stamatakis H, Tsiklakis K. Evaluation of the severity of temporomandibular joint osteoarthritic changes related to age using cone beam computed tomography. Dentomaxillofac Radiol 2009;38:141-7.	170 (12,14)	197 (14,07)
37	Stratemann SA, Huang JC, Maki K, Miller AJ, Hatcher DC. Comparison of cone beam computed tomography imaging with physical measures. Dentomaxillofac Radiol 2008;37:80-93.	170 (11,33)	185 (12,33)
38	Tuzoff DV, Tuzova LN, Bornstein MM, Krasnov AS, Kharchenko MA, Nikolenko SI, et al. Tooth detection and numbering in panoramic radiographs using convolutional neural networks. Dentomaxillofac Radiol 2019;48:20180051.	167 (41)	196 (49)
39	Horner K, Islam M, Flygare L, Tsiklakis K, Whaites E. Basic principles for use of dental cone beam computed tomography: consensus guidelines of the European Academy of Dental and Maxillofacial Radiology. Dentomaxillofac Radiol 2009;38:187-95.	161 (11,5)	192 (13,71)
40	Bedogni A, Blandamura S, Lokmic Z, Palumbo C, Ragazzo M, Ferrari F, et al. Bisphosphonate-associated jawbone osteonecrosis: a correlation between imaging techniques and histopathology. Oral Surg Oral Med Oral Pathol Oral Radiol Endod 2008;105:358-64.	157 (10,47)	167 (11,13)
41	Tyndall DA, Brooks SL. Selection criteria for dental implant site imaging: a position paper of the American Academy of Oral and Maxillofacial radiology. Oral Surg Oral Med Oral Pathol Oral Radiol Endod 2000;89:630-7.	157 (6,83)	207 (9)
42	Suomalainen A, Vehmas T, Kortesniemi M, Robinson S, Peltola J. Accuracy of linear measurements using dental cone beam and conventional multislice computed tomography. Dentomaxillofac Radiol 2008;37:10-7.	155 (10,33)	173 (11,53)
43	Ruttimann UE, Webber RL, Hazelrig JB. Fractal dimension from radiographs of peridental alveolar bone. A possible diagnostic indicator of osteoporosis. Oral Surg Oral Med Oral Pathol 1992;74:98-110.	154 (4,97)	162 (5,23)
44	Katsumata A, Fujishita M, Maeda M, Ariji Y, Ariji E, Langlais RP. 3D-CT evaluation of facial asymmetry. Oral Surg Oral Med Oral Pathol Oral Radiol Endod 2005;99:212-20.	153 (8,5)	179 (9,94)
45	Webber RL, Horton RA, Tyndall DA, Ludlow JB. Tuned-aperture computed tomography (TACT). Theory and application for three-dimensional dento-alveolar imaging. Dentomaxillofac Radiol 1997;26:53-62.	148 (5,69)	158 (6,08)
46	Taguchi A, Suei Y, Ohtsuka M, Otani K, Tanimoto K, Ohtaki M. Usefulness of panoramic radiography in the diagnosis of postmenopausal osteoporosis in women. Width and morphology of inferior cortex of the mandible. Dentomaxillofac Radiol 1996;25:263-7.	146 (5,41)	164 (6,07)
47	Farman AG. ALARA still applies. Oral Surg Oral Med Oral Pathol Oral Radiol Endod 2005;100:395-7.	145 (8,06)	157 (8,73)
48	de Oliveira AE, Cevidanes LH, Phillips C, Motta A, Burke B, Tyndall D. Observer reliability of three-dimensional cephalometric landmark identification on cone-beam computerized tomography. Oral Surg Oral Med Oral Pathol Oral Radiol Endod 2009;107:256-65.	144 (10,26)	158 (11,29)
49	Mischkowski RA, Pulsfort R, Ritter L, Neugebauer J, Brochhagen HG, Keeve E, et al. Geometric accuracy of a newly developed cone-beam device for maxillofacial imaging. Oral Surg Oral Med Oral Pathol Oral Radiol Endod 2007;104:551-9.	144 (9)	157 (9,81)
50	Ledgerton D, Horner K, Devlin H, Worthington H. Radiomorphometric indices of the mandible in a British female population. Dentomaxillofac Radiol 1999;28:173-81.	144 (6)	163 (6,79)
51	Marmulla R, Wörtche R, Mühling J, Hassfeld S. Geometric accuracy of the NewTom 9000 Cone Beam CT. Dentomaxillofac Radiol 2005;34:28-31.	141 (7,83)	164 (9,11)
52	Chiandussi S, Biasotto M, Dore F, Cavalli F, Cova MA, Di Lenarda R. Clinical and diagnostic imaging of bisphosphonate-associated osteonecrosis of the jaws. Dentomaxillofac Radiol 2006;35:236-43.	138 (8,18)	163 (9,59)
53	Mouyen F, Benz C, Sonnabend E, Lodter JP. Presentation and physical evaluation of Radio VisioGraphy. Oral Surg Oral Med Oral Pathol 1989;68:238-42.	135 (3,97)	159 (4,68)
54	Zinser MJ, Mischkowski RA, Sailer HF, Zöller JE. Computer-assisted orthognathic surgery: feasibility study using multiple CAD/CAM surgical splints. Oral Surg Oral Med Oral Pathol Oral Radiol 2012;113:673-87.	132 (12)	146 (13,27)
55	Larheim TA, Abrahamsson AK, Kristensen M, Arvidsson LZ. Temporomandibular joint diagnostics using CBCT. Dentomaxillofac Radiol 2015;44:20140235.	131 (16,38)	156 (19,5)
56	Kumar V, Ludlow JB, Mol A, Cevidanes L. Comparison of conventional and cone beam CT synthesized cephalograms. Dentomaxillofac Radiol 2007;36:263-269.	131 (8,19)	146 (9,13)
57	Cavalcanti MG, Rocha SS, Vannier MW. Craniofacial measurements based on 3D-CT volume rendering: implications for clinical applications. Dentomaxillofac Radiol 2004;33:170-6.	131 (6,89)	148 (7,79)
58	Liu DG, Zhang WL, Zhang ZY, Wu YT, Ma XC. Localization of impacted maxillary canines and observation of adjacent incisor resorption with cone-beam computed tomography. Oral Surg Oral Med Oral Pathol Oral Radiol Endod 2008;105:91-8.	130 (8,67)	145 (9,67)
59	Katsumata A, Hirukawa A, Okumura S, Naitoh M, Fujishita M, Ariji E, et al. Effects of image artifacts on gray-value density in limited-volume cone-beam computerized tomography. Oral Surg Oral Med Oral Pathol Oral Radiol Endod 2007;104:829-36.	130 (8,13)	148 (9,25)
60	Hildebolt CF. Osteoporosis and oral bone loss. Dentomaxillofac Radiol 1997;26:3-15.	128 (4,92)	138 (5,31)
61	Carter L, Farman AG, Geist J, Scarfe WC, Angelopoulos C, Nair MK, et al. American Academy of Oral and Maxillofacial Radiology executive opinion statement on performing and interpreting diagnostic cone beam computed tomography. Oral Surg Oral Med Oral Pathol Oral Radiol Endod 2008;106:561-2.	126 (8,4)	150 (10)
62	Metzger MC, Hohlweg-Majert B, Schwarz U, Teschner M, Hammer B, Schmelzeisen R. Manufacturing splints for orthognathic surgery using a three-dimensional printer. Oral Surg Oral Med Oral Pathol Oral Radiol Endod 2008;105:e1-7.	126 (8,4)	145 (9,67)
63	Honda K, Larheim TA, Maruhashi K, Matsumoto K, Iwai K. Osseous abnormalities of the mandibular condyle: diagnostic reliability of cone beam computed tomography compared with helical computed tomography based on an autopsy material. Dentomaxillofac Radiol 2006;35:152-7.	125 (7,35)	142 (8,35)
64	Jacobs R, Mraiwa N, vanSteenberghe D, Gijbels F, Quirynen M. Appearance, location, course, and morphology of the mandibular incisive canal: an assessment on spiral CT scan. Dentomaxillofac Radiol 2002;31:322-7.	125 (5,95)	152 (7,24)
65	Goldman M, Pearson AH, Darzenta N. Reliability of radiographic interpretations. Oral Surg Oral Med Oral Pathol 1974;38:287-93.	124 (2,53)	151 (3,08)
66	Araki K, Maki K, Seki K, Sakamaki K, Harata Y, Sakaino R, et al. Characteristics of a newly developed dentomaxillofacial X-ray cone beam CT scanner (CB MercuRay): system configuration and physical properties. Dentomaxillofac Radiol 2004;33:51-9.	123 (6,47)	142 (7,47)
67	Wenzel A. Digital radiography and caries diagnosis. Dentomaxillofac Radiol 1998;27:3-11.	123 (4,92)	132 (5,28)
68	Kramer RM, Williams AC. The incidence of impacted teeth. A survey at Harlem hospital. Oral Surg Oral Med Oral Pathol 1970;29:237-41.	123 (2,32)	152 (2,87)
69	Larheim TA. Current trends in temporomandibular joint imaging. Oral Surg Oral Med Oral Pathol Oral Radiol Endod. 1995;80:555-76.	122 (4,36)	135 (4,82)
70	Hung K, Montalvao C, Tanaka R, Kawai T, Bornstein MM. The use and performance of artificial intelligence applications in dental and maxillofacial radiology: A systematic review. Dentomaxillofac Radiol 2020;49:20190107.	121 (40,33)	134 (44,67)
71	Loubele M, Van Assche N, Carpentier K, Maes F, Jacobs R, van Steenberghe D, et al. Comparative localized linear accuracy of small-field cone-beam CT and multislice CT for alveolar bone measurements. Oral Surg Oral Med Oral Pathol Oral Radiol Endod 2008;105:512-8.	121 (8,07)	150 (10)
72	Bollen AM, Taguchi A, Hujoel PP, Hollender LG. Case-control study on self-reported osteoporotic fractures and mandibular cortical bone. Oral Surg Oral Med Oral Pathol Oral Radiol Endod. 2000;90:518-24.	121 (5,27)	138 (6)
73	Gröndahl HG, Gröndahl K, Webber RL. A digital subtraction technique for dental radiography. Oral Surg Oral Med Oral Pathol 1983;55:96-102.	121 (3,03)	149 (3,73)
74	Panchbhai AS. Dental radiographic indicators, a key to age estimation. Dentomaxillofac Radiol 2011;40:199-212.	120 (10)	155 (12,92)
75	Ariji Y, Kuroki T, Moriguchi S, Ariji E, Kanda S. Age changes in the volume of the human maxillary sinus: a study using computed tomography. Dentomaxillofac Radiol 1994;23:163-8.	120 (4,14)	135 (4,66)
76	De Lange J, Van den Akker HP. Clinical and radiological features of central giant-cell lesions of the jaw. Oral Surg Oral Med Oral Pathol Oral Radiol Endod 2005;99:464-70.	118 (6,56)	153 (8,5)
77	Hwang JJ, Jung YH, Cho BH, Heo MS. An overview of deep learning in the field of dentistry. Imaging Sci Dent 2019;49:1-7.	117 (29,25)	138 (34,5)
78	Loubele M, Maes F, Schutyser F, Marchal G, Jacobs R, Suetens P. Assessment of bone segmentation quality of cone-beam CT versus multislice spiral CT: a pilot study. Oral Surg Oral Med Oral Pathol Oral Radiol Endod 2006;102:225-34.	117 (6,88)	136 (8)
79	Pontual MLD, Freire JSL, Barbosa JMN, Frazão MAG, Pontual AD. Evaluation of bone changes in the temporomandibular joint using cone beam CT. Dentomaxillofac Radiol 2012;41:24-9.	116 (10,55)	130 (11,82)
80	Gröndahl HG, Gröndahl K. Subtraction radiography for the diagnosis of periodontal bone lesions. Oral Surg Oral Med Oral Pathol 1983;55:208-13.	114 (2,85)	125 (3,13)
81	Brüllmann D, Schulze RK. Spatial resolution in CBCT machines for dental/maxillofacial applications-what do we know today? Dentomaxillofac Radiol 2015;44:20140204.	113 (14,13)	123 (15,36)
82	Vandenberghe B, Jacobs R, Yang J. Detection of periodontal bone loss using digital intraoral and cone beam computed tomography images: an in vitro assessment of bony and/or infrabony defects. Dentomaxillofac Radiol 2008;37:252-60.	113 (7,53)	129 (8,6)
83	Su L, Weathers DR, Waldron CA. Distinguishing features of focal cemento-osseous dysplasia and cemento-ossifying fibromas. II. A clinical and radiologic spectrum of 316 cases. Oral Surg Oral Med Oral Pathol Oral Radiol Endod 1997;84:540-9.	113 (4,35)	158 (6,08)
84	Eberhardt JA, Torabinejad M, Christiansen EL. A computed tomographic study of the distances between the maxillary sinus floor and the apices of the maxillary posterior teeth. Oral Surg Oral Med Oral Pathol 1992;73:345-6.	113 (3,65)	138 (4,45)
85	Bernardes RA, de Moraes IG, Húngaro Duarte MA, Azevedo BC, de Azevedo JR, Bramante CM. Use of cone-beam volumetric tomography in the diagnosis of root fractures. Oral Surg Oral Med Oral Pathol Oral Radiol Endod 2009;108:270-7.	112 (8)	137 (9,79)
86	Maeda M, Katsumata A, Ariji Y, Muramatsu A, Yoshida K, Goto S, et al. 3D-CT evaluation of facial asymmetry in patients with maxillofacial deformities. Oral Surg Oral Med Oral Pathol Oral Radiol Endod 2006;102:382-90.	111 (6,53)	130 (7,65)
87	Ritter L, Lutz J, Neugebauer J, Scheer M, Dreiseidler T, Zinser MJ, et al. Prevalence of pathologic findings in the maxillary sinus in cone-beam computerized tomography. Oral Surg Oral Med Oral Pathol Oral Radiol Endod 2011;111:634-40.	110 (9,17)	98 (8,17)
88	Nkenke E, Zachow S, Benz M, Maier T, Veit K, Kramer M, et al. Fusion of computed tomography data and optical 3D images of the dentition for streak artefact correction in the simulation of orthognathic surgery. Dentomaxillofac Radiol 2004;33:226-32.	110 (5,79)	123 (6,47)
89	Kruger E, Thomson WM, Konthasinghe P. Third molar outcomes from age 18 to 26: findings from a population-based New Zealand longitudinal study. Oral Surg Oral Med Oral Pathol Oral Radiol Endod 2001;92150-5.	110 (5)	140 (6,36)
90	de Leeuw R, Boering G, Stegenga B, de Bont LG. Radiographic signs of temporomandibular joint osteoarthrosis and internal derangement 30 years after nonsurgical treatment. Oral Surg Oral Med Oral Pathol Oral Radiol Endod 1995;79:382-92.	110 (3,93)	117 (4,18)
91	Devlin H, Karayianni K, Mitsea A, Jacobs R, Lindh C, van der Stelt P, et al. Diagnosing osteoporosis by using dental panoramic radiographs: the OSTEODENT project. Oral Surg Oral Med Oral Pathol Oral Radiol Endod 2007;104:821-8.	109 (6,81)	132 (8,25)
92	Mraiwa N, Jacobs R, Van Cleynenbreugel J, et al. The nasopalatine canal revisited using 2D and 3D CT imaging. Dentomaxillofac Radiol 2004;33:396-402.	109 (5,74)	122 (6,42)
93	Draenert FG, Coppenrath E, Herzog P, Müller S, Mueller-Lisse UG. Beam hardening artefacts occur in dental implant scans with the NewTom cone beam CT but not with the dental 4-row multidetector CT. Dentomaxillofac Radiol. 2007;36:198-203.	108 (6,75)	125 (7,81)
94	Aydin U, Yilmaz HH, Yildirim D. Incidence of canine impaction and transmigration in a patient population. Dentomaxillofac Radiol 2004;33:164-9.	108 (5,68)	131 (6,89)
95	Hiraiwa T, Ariji Y, Fukuda M, Kise Y, Nakata K, Katsumata A, Fujita H, Ariji E. A deep-learning artificial intelligence system for assessment of root morphology of the mandibular first molar on panoramic radiography. Dentomaxillofac Radiol 2019;48:20180218.	107 (26,75)	126 (31,5)
96	Friedlander AH, Lande A. Panoramic radiographic identification of carotid arterial plaques. Oral Surg Oral Med Oral Pathol 1981;52:102-104.	107 (2,58)	123 (2,93)
97	Naitoh M, Hiraiwa Y, Aimiya H, Gotoh K, Ariji E. Accessory mental foramen assessment using cone-beam computed tomography. Oral Surg Oral Med Oral Pathol Oral Radiol Endod 2009;107:289-94.	106 (7,57)	122 (8,71)
98	Yaşar F, Akgünlü F. The differences in panoramic mandibular indices and fractal dimension between patients with and without spinal osteoporosis. Dentomaxillofac Radiol 2006;35:1-9.	106 (6,24)	120 (7,06)
99	Kawamata A, Fujishita M, Ariji Y, Ariji E. Three-dimensional computed tomographic evaluation of morphologic airway changes after mandibular setback osteotomy for prognathism. Oral Surg Oral Med Oral Pathol Oral Radiol Endod. 2000;89:278-87.	109 (4,74)	123 (5,35)
100	Farman AG, Farman TT. A comparison of 18 different x-ray detectors currently used in dentistry. Oral Surg Oral Med Oral Pathol Oral Radiol Endod 2005;99:485-9.	105 (5,83)	126 (7)
101	Katzberg RW, Schenck J, Roberts D, Tallents RH, Manzione JV, Hart HR, Foster TH, Wayne WS, Bessette RW. Magnetic resonance imaging of the temporomandibular joint meniscus. Oral Surg Oral Med Oral Pathol 1985;59:332-5.	105 (2,76)	86 (2,26)

**Table 2:** Countries, institutions of origin and authors with four or more top cited articles in dental radiology journals.

Category	Description	Publication Number
Country	USA	40
	Japan	13
	Germany	12
	Belgium	5
	Brazil	4
	United Kingdom	3
Institution	Univ. of North Carolina	11
	Catholic Univ. Leuven	3
	Aichi Gakuin Univ.	3
	Asahi Univ.	3
	Nihon Univ.	3
	Univ. of Cologne	3
	Univ. of Louisville	3
	Univ. of Manchester	3
	Univ. of Michigan	3
	Univ. of São Paulo	3
Authors	Ludlow JB	8
	Ariji E	6
	Jacobs R	6
	Ariji Y	4
	Brooks SL	4
	Katsumata A	4

**Table 3:** Radiographic technique and research topics of 100 top cited papers.

Category	Description	Publication Number
Radiographic Technique	Mixed techniques	38
	CBCT	29
	Panoramic radiographs	10
	CT	6
	Intraoral radiographs	5
	3D-CT	4
	Digital radiography	2
	Radiation therapy	2
	Other (each with n≤1)	4
Research topic		
Diagnosis (40%)	Osteoporosis	10
	Orthodontics	8
	Implantology	8
	Caries	3
	Endodontology	3
	Bisphosphonate-related osteonecrosis of the jaws	3
	Oral pathology	3
	Periodontology	2
Image analysis (27%)	TMJ imaging	9
	Anatomic landmarks	7
	Artifacts	5
	Reliability	3
	Hounsfield units	2
	Reproducibility	1
Dose & geometric measurements (23%)	Dose measurements	12
	Linear measurements	11
New techniques (10%)	New techniques	10

## Data Availability

The datasets used and/or analyzed during the current study are available from the corresponding author.
